# *UKuvala umkhokha* ‘ending the curse’: Unpacking the healing practice steps followed by traditional healers in KwaZulu-Natal when working with rape survivors

**DOI:** 10.4102/sajpsychiatry.v32i0.2645

**Published:** 2026-06-01

**Authors:** Nqobile Muthwa, Yaseen Ally

**Affiliations:** 1 Department of Psychology, Faculty of Health Sciences, Nelson Mandela University, Port Elizabeth, South Africa; 2 Department of Psychology, Ngwelezane Hospital, Empangeni, South Africa; 3 Department of Psychology, College of Human Sciences, University of South Africa, Pretoria, South Africa

**Keywords:** medical pluralism, rape, sexual violence, traditional health practitioners (THP), post-rape care, spirituality

## Abstract

**Background:**

South Africa is considered as the epicentre of rape. Despite the establishment of hospital-based post-rape centres, uptake remains limited. Within the context of medical pluralism, many individuals utilise both biomedical and traditional healing systems, which are often perceived as culturally congruent and accessible. This study explored post-rape care services provided by traditional health practitioners (THPs) when survivors seek care outside the formal healthcare system.

**Aim:**

The study aimed to demystify traditional healing methods used in post-rape care by describing treatment approaches and formulations.

**Setting:**

The study was conducted in KwaZulu-Natal, South Africa, which is a province with high rates of gender-based violence and rape.

**Methods:**

A qualitative descriptive design was used. Fifteen THPs, including faith healers, diviners, and herbalists, participated in one-on-one interviews conducted in isiZulu and translated into English. Data were analysed using thematic analysis and the healthworlds framework.

**Results:**

Findings indicated that rape was often understood as spiritual contamination affecting both survivors and their bloodline. Healing practices therefore focused on addressing this perceived spiritual transgression.

**Conclusion:**

The results of this study highlight the importance of culture and spirituality in shaping understandings of rape, treatment formulation, and holistic post-rape care.

**Contribution:**

The study demonstrates the significance of traditional healing methods in providing holistic post-rape care.

## Introduction

Rape is recognised as a human rights violation, adversely affecting millions of women globally.^1,2^ South Africa is widely recognised as having the highest incidence of reported rape cases globally, leading to its status as the ‘rape capital’ of the world.^3^ Rape has immediate and long-lasting physiological and psychological morbidities.^4^ Rape survivors need to access an array of services following rape, such as emergency contraception, termination of pregnancy (TOP), mental health services and access to post-exposure prophylaxis (PEP). To improve access to care, Thuthuzela Care Centres were developed in all nine provinces.^5^ These centres are linked to government hospitals and provide care to survivors of rape in an environment that is meant to reduce secondary trauma.^5^ Despite the integration of post-rape care services in the formal healthcare system, the utilisation and adherence to these services remain poor.^6^

In South Africa, there exists medical pluralism where individuals engage with multiple health systems. Often, individuals will engage with biomedical health systems in conjunction with traditional healing methods in comprehending their health presentations and in the pursuit of health. It is estimated that over 80% of South Africans consult with traditional healers for their health needs.^7^ What constitutes post-rape care outside the formal healthcare system and the services provided by traditional health practitioners following rape remain veiled in mystery. It is crucial to understand health provision in the context of rape, as appropriate post-rape care addresses the immediate and long-term psychological and physical needs of rape survivors. This study aimed to understand treatment methods employed by traditional health practitioners in their management of rape survivors in a country marred by sexual trauma.

## Research methods and design

### Study design

The research employed a qualitative and descriptive design, situated under a social constructionist paradigm. The theoretical underpinnings of this study were informed by the healthworlds framework. The healthworlds framework focuses on how health and illness are as much a biological phenomenon as it is socially constructed. Within this framework, understanding of health, illness, health-seeking behaviours and the acceptability of care are produced and negotiated within specific cultural and social contexts.^8^ This means that how traditional health practitioners make sense of their role as service providers in post-rape care will be affected by not only the psychological or medical needs of rape survivors but also by how the larger society shapes what rape is.

### Study setting

The study was conducted in the KwaZulu-Natal (KZN) Province, South Africa. This province was selected because of its notably high incidence of gender-based violence and rape cases.^9^ Crime in KZN is often characterised by violence, with rape survivors often sustaining physical injuries during the assault. Women in KwaZulu-Natal are more likely to be raped by unknown individuals, experience rape in their own homes and are more prone to experiencing physical violence, which exacerbates their symptoms.^10^ Additionally, factors such as higher rates of poverty and lower educational status are more prevalent in KZN compared to other provinces in South Africa.^11^ All these factors intersect and create a heightened vulnerability for women in KZN to the development of psychopathology following rape.^12^

### Sampling

A purposive sampling approach was used to facilitate the intentional selection of participants.^13,14^ The study targeted traditional healers who were over the age of 18; participants had to belong to one of the three healer categories and identify as either a *sangoma* [diviner], *umthandazi* [faith healer] or *inyanga* [herbalist]. Additionally, participants were required to report having at least 2 years of work experience providing treatment for survivors of rape. Participants had to have completed the required initiation and training rites prior to participating in the study.

Participants were recruited through African traditional churches, traditional pharmacies, Facebook groups focused on traditional healing and community notice boards. A total of 33 traditional healers were screened, and a total of 15 were eligible to participate in the study. The excluded participants included seven traditional healers excluded for having limited experience working with rape survivors. Six healers were excluded for having less than 2 years of experience working as traditional healers. Four healers were identified as self-initiates and had not undergone formal initiation and training rites. One healer was excluded for not granting permission to record the interview. Participants ages varied from 19 years old to 65 years old. Thirteen of the healers worked full time as traditional healers, and two worked as traditional health practitioners (THPs) part time. The demographic profile of the participants is presented in Table 1.

**TABLE 1:** Demographic information table.

Name	Gender	Age (years)	Years of practice as a THP	Traditional healer identification category	Geographic segmentation
Dabula	Male	33	7	Sangoma / Umfembi	Peri-urban
Sbu	Male	36	15	Inyanga	Peri-urban
Mam Thobile	Female	56	15	Umthandazi	Peri-urban
Vusabadala	Female	19	3	Sangoma / Umthandazi	Rural
Mantuli	Female	45	4	Sangoma / Umthandazi/ Inyanga	Peri-urban
Mkhulu Ndawana	Male	46	20	Sangoma/ Inyanga/ Umthandazi	Rural
Bab’ Skhumba	Male	39	10	Sangoma / Umthandazi	Rural
Bab’ Bhebhe	Male	38	> 10	Sangoma / Inyanga	Rural
Khuzamazulu	Male	65	32	Sangoma	Rural
Mlomo ongathethi manga	Male	39	4	Sangoma / Umthandazi	Rural
Mkhulu Shinga	Male	41	5	Sangoma / Inyanga	Rural
Makhosazane	Female	48	> 10	Umthandazi / Inyanga	Peri-urban
Makhosi Dludla	Female	26	5	Umthandazi	Urban
Nhlanhla	Male	32	4	Umthandazi	Peri-urban
Mam Dlomo	Female	55	17	Sangoma / Umthandazi	Urban

THP, traditional health practitioner.

### Data collection

A combination of face-to-face as well as telephone interviews was conducted. The face-to-face interviews were conducted in traditional pharmacies, participants’ homes and in their workspaces, including *indumba*, a traditional hut that serves as the primary space for consultation, divination and healing practices. Participants were asked semi-structured, open-ended questions exploring their experiences of working with rape survivors. Questions focused on when survivors typically present for consultation, the concerns they commonly present with, how participants understand and define rape, and the forms of treatment or healing practices they provide. Interviews lasted approximately 1 h. All 15 participants requested to have the interviews conducted in isiZulu.

### Data analysis

A thematic analysis was conducted following the stages outlined by Braun et al.^15^ These included going over the transcripts several times to make sense of emerging meaning and patterns. Initial codes were generated, and extracts from each interview were grouped under the corresponding codes. Related codes were grouped together, and initial themes were developed. The themes were reviewed, some merged with others, some were collapsed. The themes were refined against the data. What each theme meant in relation to the study was explored, final themes and subthemes were made. The results are presented in a descriptive manner with the aim of providing a systematic account of care provision.

### Ethical considerations

Ethical clearance was received from the Nelson Mandela University Research Ethics Committee (human) (protocol number: H22-HEA-PSY-001). Participants were given information sheets prior to participation. Participants signed informed consent forms, as well as forms requesting access to their homes or workspace when indicated. For confidentiality, anonymity was maintained by using pseudonyms during the interviews, transcription period and during data presentation.

## Results

**FIGURE 1:**
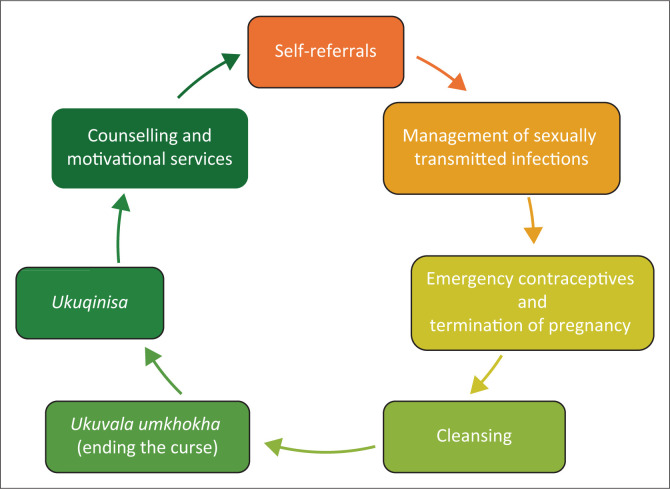
Illustration of treatment cycle.

The analysis identified several themes describing how traditional health practitioners manage rape within their healing practices. These themes collectively represent a treatment cycle that begins with pathways to disclosure and the management of physical consequences of rape, followed by spiritually oriented interventions and psychosocial support. This treatment cycle is illustrated in Figure 1.

### Theme 1: Referral pathways and rape disclosure

The study revealed a lack of established referral pathways that exist for rape survivors to access the services of traditional health practitioners following rape. Two distinct pathways were identified by participants, which resulted in individuals receiving care. The first pathway to care involved self-referrals, where individuals, aware of the cultural significance of traditional healing, sought the services of a traditional healer following a rape incident:

‘It differs how can I put in Zulu on the circumstances you get me. One would just reveal it and then one would be afraid because they don’t want to be judged or they don’t want you to look at them differently … Sometimes they would just tell you straight because they just want things to cleanse and purify themselves so yah.’ (Male, 36 years, Inyanga)

The second pathway involved individuals seeking consultations for various ailments or troubles, including but not limited to misfortune, interpersonal challenges or financial hardships. During the divination process, a history of rape would often be revealed:

‘Those that have come to me, there are those that come because they are having problems in their lives. In our talks and consultation, we find that there is a story like she had an abortion, and that child is now giving her problems. And she will explain she was raped and got pregnant then aborted it.’ (Female, 56 years, Umthandazi)

Another pathway used by traditional healers to identify a history of rape was sensing the distress of others. Healers described having the ability to connect, sense and feel the emotions of others:

‘You feel the emotions of that person here for consultation at that time. If they had been in that situation they would never tell unless you feel her emotions or you feel her cry, you feel the pain she is feeling at that time, all you can do is to speak to the person in the way that is appropriate so that she can open up and come back to get help going forward.’ (Female, 26 years, Umthandazi)

Healers would then ask questions around what was causing the individual distress, and then rely on the rape victim disclosing that they have been sexually assaulted and then treatment would be based on the disclosure. Subsequently, individuals would undergo treatment for the rape based on the guidance provided by the healer’s spiritual guides.

### Theme 2: Management of the physical consequences of rape

#### Sexually transmitted infections

The management of sexually transmitted infections (STIs) following rape is crucial for the overall health of the victim, as untreated STIs can have long-lasting consequences on health.^16^ The management of STIs was also an important part of treatment for those who presented to traditional healers shortly after being sexually assaulted. Opinions varied among healers regarding who should manage STIs. Some healers advocated for immediate referral to biomedical practitioners for STI management, believing that biomedical care was essential immediately after rape:

‘As I had explained before, we traditional healers must report this to the western clinics, as there are certain things that we don’t have, like testing kits. She must then start by going to the clinic and run tests, then after that is where we provide her with certain traditional medicine for cleaning the genital area.’ (Male, 39 years, Sangoma/Umthandazi)

On the other hand, some healers believed that traditional healers should handle STI management instead of referring patients to biomedical practitioners:

‘We heal them here, we give them herbs that they can use as enemas, some they drink and some they use to wash the genital area to make sure that all the dirt and infections come out.’ (Female, 55 years, Sangoma/Umthandazi)

There was, however, a common belief among most healers that certain STIs would not resolve despite rape survivors receiving treatment for them in biomedical care settings. They believed that specific traditional herbs or rituals were necessary for the complete resolution of these infections:

‘Usually they would say, they don’t feel well in the private part, they are having a discharge, there is a smell, the private part is always sore, some will even be afraid to have sex even if they were raped at a young age and they would not want to have sex and they have some infection. And when the doctors have failed, we try our own way, mix herbs that she baths and drinks with to kill the bacteria.’ (Female, 56 years, Umthandazi)

The above extracts highlight that traditional healers are aware of the high risk of contracting STIs and human immunodeficiency virus (HIV) following rape. They also showed that traditional healers were often open to joint multidisciplinary management with Western practitioners in managing these symptoms.

#### Emergency contraception and termination of pregnancy

Survivors of rape are at a greater risk of unwanted pregnancies, as rapists often fail to use condoms. Emergency contraceptives are essential in post-rape care as they prevent the burden of rape-related pregnancies, which often have devastating physical and psychological effects on the victim.^16^ Should emergency contraception fail or not be administered timeously; healthcare providers need to give survivors the option of terminating pregnancy.^17^ There were opposing views among healers regarding the provision of emergency contraception and TOP. Healers who worked exclusively with herbs and did not possess any connection to spiritual elements were more inclined to provide such services:

‘And the sperm of a man in the womb that is unnecessary from the perpetrator. Because you can’t keep a child from … the rape child, so, we also help them clean their womb so that they don’t fall pregnant out of rape … We ask you whether you want to keep that child or not, if you don’t want to keep the child, we give you herbs that are organic and not harmful to you.’ (Male, 36 years, Inyanga)

Conversely, healers whose spiritual guides were at the forefront of their healing practices often opposed providing such services. The following extract is from a *sangoma* and represents the opposition to providing such services that was seen in the *abathandazi* (plural) and *izangoma* (plural) groups:

‘We cannot help you with anything there, it’s the western doctors who can help you and give you pills to kill that child. We don’t have medication that kills, all the medicine you are seeing here is edible it doesn’t kill, you can tell us but we won’t help you.’ (Male, 65 years, Sangoma)

Although most *sangomas* and *abathandazi* did not express opposition to the TOP services, they did not offer such services, but rather sent patients who required such services to biomedical service providers.

### Theme 3: Spiritual cleansing

The cleansing process highlighted the inherent spiritual and cultural beliefs that are ingrained in African communities. Most events are not only viewed as occurring on the physical plane but also have spiritual significance. Spiritual cleansing was believed to play a role in not only healing the individual psychologically but also tapping into and healing their ancestral line and healing them spiritually. Cleansing played the most paramount role in the treatment process, as there was a belief that rape is as much of a physical occurrence as it is a spiritual violation. Rape was constructed as an act that leads to the formation of *ukungcola* [impurity] or *amathunzi amnyama* [dark shadows]. Cleaning became an essential part of treatment as it removed this spiritual contamination:

‘How do you cleanse that person? You take her to the flowing river, or you go to the ocean and that person must bring meat or dung and a white chicken. Why dung or dirty meat? It is to cleanse that shadow. Just like when one has had a family member that has passed on, they cleanse with that to remove that shadow. So, then there’s a session where she cleanses with the white chicken, why does she cleanse with that? She’s cleansing the ancestors that were stained and insulted by that rapist, being raped is being insulted.’ (Female, 45 years, Sangoma/Umthandazi/Inyanga)

Dark shadows were described as negative energy that attaches itself to the individual following rape, resulting in a host of negative outcomes for the individual. Rape was perceived to not only affect the individual’s spiritual wellbeing but also to violate the wellbeing of one’s ancestors. Spiritual cleansing was therefore essential in restoring balance and in countering the spiritual consequences of rape.

### Theme 4: Ukuvala umkhokha or ending the curse

After the cleansing process, the next step was ukuvala umkhokha or ‘ending the curse’. There was a belief that rape not only led to the development of negative spiritual attachments, but it also resulted in a curse that not only affected the individual but also tainted those who are living in the individual’s lineage. This curse led to the family and the victim being predisposed to future rape experiences. During or after the cleansing process, healers would then close this intergenerational curse to prevent the victim from being prone to repeated rape and to prevent other family members from being subjected to rape:

‘No, it is not over, this thing needs to be blocked so that this never happens again. The rape thing, once it has happened and it wasn’t blocked it might happen again.’ (Male, 39 years, Sangoma/Umthandazi)

The current rape itself could be attributed to an outcome of this curse if there was a history of rape in the family and the curse was not blocked. In order to prevent repeated sexual trauma, ukuvala umkhokha plays a crucial step in the healing process and in managing long-term psychological outcomes for rape survivors.

### Theme 5: Ukuqinisa – Strengthening and protection

Some of the healers interviewed in the study highlighted the importance of *ukuqinisa*, which is a preventative procedure which includes creating incisions in the individual’s body and then applying herbs subcutaneously. The process was often conducted after the cleansing process if issues relating to witchcraft were identified during the divination process, as attracting negative energies for the individual. The process was meant to eliminate negative energies but also make the individual less susceptible to witchcraft:

‘When a person is given strength, there are lot of things that a healer will work with. But a person is strengthened so that when others do bad things, those things do not bad affect or come to the person easily. It is done so that bad spirits do not reach a person while they are weak. It is done so that they are not easily hit by bad things.’ (Male, 33 years, Sangoma/Umfembi)

If there was a belief that witchcraft was at the forefront of the individuals’ misfortunes, then most healers reported that they would then strengthen the individual to prevent further harm from befalling the individual.

### Theme 6: Counselling and motivation

The final step of the healing process included the provision of counselling services. There was consensus among the traditional healers around the need to provide some form of counselling or motivation to manage issues such as loss of self-esteem. There were, however, opposing opinions on how counselling services should be rendered as well as on who should provide the services. Some healers believed that traditional healers were equipped to provide counselling:

‘I do the counselling as a traditional healer because I will be assisted by my ancestors, I don’t just talk, whatever I’m saying doesn’t come from me and my knowledge but I’m with my ancestors that when they possess you no matter how young you are, you get the wisdom of an elderly person.’ (Male, 46 years, Sangoma/Inyanga/Umthandazi)

In contrast, some healers believed that providing counselling services was beyond the scope of THPs and that rape survivors needed to be referred to mental health professionals:

‘That situation needs what we call counselling. We as traditional healers are not much involved in that because we don’t have a herb that can help with that, that person needs professional psychologist that knows how to deal with that situation.’ (Male, 39 years, Sangoma/Umthandazi)

When counselling was provided by traditional healers, it often took the form of motivational guidance aimed at restoring hope and resilience rather than addressing clinical psychological symptoms:

‘We don’t just end by treating them, but we sit down and counsel them and give them hope and also make examples that I’ve been there, other women have been there, and this is how I moved on from it. You need to find a way to bring her back.’ (Female, 19 years, Sangoma/Umthandazi)

These findings suggest that while counselling is recognised as an important component of post-rape care, there is variation among traditional healers regarding their role in providing psychological support.

### Theme 7: Ambiguity in termination of services

The point of termination was ambiguous as some healers ended their services after the cleansing process, where both the patient and their ancestors were cleansed of the rape violation. This was believed to be a restorative process psychologically in addition to its ability to prevent future calamities while attracting luck in the individual’s life. Several healers spoke about the importance of referring their patients to access counselling services from mental health practitioners. It was unclear if, after this referral was made, there was still a need for the patient to come back for services with the traditional healer. Those who provided counselling services themselves spoke about allocating time to provide survivors with this service. There was a lack of consensus on whether the service was rendered during the cleansing process or whether it was a stand-alone service that individuals would exclusively attend. There was also no consensus regarding what the markers were for symptom remission, which would be considered as a criterion for the termination of services. This situation leaves opportunities for gaps in treatment and prevents further referrals from being made when necessary.

## Discussion

The results of the study illustrated that in the healthworld of traditional healers’ rape is not only perceived to be a physical and psychological violation but also a spiritual injury. Within this worldview, traumatic events affecting the living are understood to extend into the spiritual realm, potentially disrupting relationships with ancestors and creating spiritual imbalance. This interpretation aligns with the healthworlds framework, which emphasises that experiences of illness and healing are shaped by socially and culturally embedded understandings of health and wellbeing.^8^ It also reflects broader evidence that THPs serve holistic roles encompassing physical, social, psychological and spiritual dimensions of illness.^18^

A central component of care was spiritual cleansing and ukuvala umkhokha [ending the curse]. Within this worldview, rape was constructed as an act that polluted and tainted the raped individual and those in their bloodline, these spiritual processes were perceived as essential in removing spiritual pollution and restoring balance between the individual, their ancestors, and the broader spiritual realm. Such interpretations reflect broader African epistemologies in which health and illness are understood holistically across physical, psychological, social, and spiritual domains.^19^

The study also highlighted structural and practice-related challenges. Firstly, the absence of formalised referral pathways between traditional healers and the biomedical health system. While some healers reported referring clients for STI testing or reproductive health services, these referrals remained informal. This finding resonates with literature on medical pluralism in South Africa, where THPs and biomedical practitioners operate in parallel with limited coordination, contributing to fragmentation and gaps in coordinated care.^20^ Secondly, the study identified a lack of uniformity in treatment approaches among THPs. Healers expressed differing views about the appropriate course of treatment in the management of STIs, reproductive health, and counselling services. This variation reflects the decentralised and diverse nature of traditional healing systems, where practices differ according to healer training, spiritual guidance, and cultural traditions.^21^

Finally, the study highlighted uncertainty regarding the termination of services. Healers were often unclear about when their involvement in a patient’s care should end, leading to ambiguities in treatment continuity. Such ambiguities lead to gaps in care, particularly in addressing long-term health needs. Overall, the findings illustrate how culturally embedded understandings of rape shape the forms of care provided by THPs. In South Africa’s pluralistic health landscape, many individuals engage with both traditional and biomedical systems. Recognising culturally grounded healing practices and addressing the lack of formal referral mechanisms may foster more coordinated and culturally responsive approaches to care that respect and integrate diverse explanatory models and practitioner roles.

### Limitations

The study was conducted in KwaZulu-Natal with predominantly Zulu participants; the findings largely reflect local cultural understandings and may differ in other contexts. Some interviews were conducted telephonically, which limited observational field notes, and the predominance of male participants may have influenced the findings, as men and women may construct rape differently. Additionally, as interviews were conducted in isiZulu, translating culturally specific expressions into English may have resulted in some loss of contextual meaning.

## Conclusion

This study explored how traditional health practitioners (THPs) in KwaZulu-Natal construct and manage cases of rape within their healing practices. The findings demonstrate that within the healthworld of THPs, rape is understood not only as a physical and psychological violation but also as a spiritual injury that requires culturally grounded forms of intervention. The study further highlights variations in treatment practices and the absence of formalised referral pathways between traditional healers and the formal healthcare system. These findings underscore the importance of recognising the role that traditional healers play in community-based responses to rape and point to the need for further research and dialogue on how culturally embedded healing systems may be considered within broader survivor care frameworks.
